# Periconceptional alcohol alters in vivo heart function in ageing female rat offspring: Possible involvement of oestrogen receptor signalling

**DOI:** 10.1113/EP090587

**Published:** 2023-03-23

**Authors:** Emily S. Dorey, John P. Headrick, Tamara M. Paravicini, Mary E. Wlodek, Karen M. Moritz, Melissa E. Reichelt

**Affiliations:** ^1^ School of Biomedical Sciences University of Queensland Brisbane Queensland Australia; ^2^ School of Pharmacy and Medical Science Griffith University Southport Queensland Australia; ^3^ School of Health and Biomedical Sciences RMIT University Melbourne Victoria Australia; ^4^ The Department of Obstetrics and Gynaecology The University of Melbourne Melbourne Victoria Australia; ^5^ Child Health Research Centre University of Queensland Brisbane Queensland Australia

**Keywords:** alcohol, cardiac development, sex differences

## Abstract

Alcohol exposure throughout gestation is detrimental to cardiac development and function. Although many women decrease alcohol consumption once aware of a pregnancy, exposure prior to recognition is common. We, therefore, examined the effects of periconceptional alcohol exposure (PC:EtOH) on heart function, and explored mechanisms that may contribute. Female Sprague–Dawley rats received a liquid diet ±12.5% v/v ethanol from 4 days prior to mating until 4 days after mating (PC:EtOH). Cardiac function was assessed via echocardiography, and offspring were culled at multiple time points for assessment of morphometry, isolated heart and aortic ring function, protein and transcriptional changes. PC:EtOH‐exposed embryonic day 20 fetuses (but not postnatal offspring) had larger hearts relative to body weight. *Ex vivo* analysis of hearts at 5–7 months old (mo) indicated no changes in coronary function or cardiac ischaemic tolerance, and apparently improved ventricular compliance in PC:EtOH females (compared to controls). At 12 mo, vascular responses in isolated aortic rings were unaltered by PC:EtOH, whilst echocardiography revealed reduced cardiac output in female but not male PC:EtOH offspring. At 19 mo, left ventricular transcript and protein for type 1 oestrogen receptor (ESR1*)*, HSP90 transcript and plasma oestradiol levels were all elevated in female PC:EtOH exposed offspring. Summarising, PC:EtOH adversely impacts in vivo heart function in mature female offspring, associated with increased ventricular oestrogen‐related genes. PC:EtOH may thus influence age‐related heart dysfunction in females through modulation of oestrogen signalling.

## Abstract (highlights)

- **What is the central question of this study?**
  What are the cardiovascular consequences of periconceptual ethanol on offspring throughout the lifespan?
- **What is the main finding and its importance?**
  It is shown for the first time that periconceptional alcohol has sex‐specific effects on heart growth, with ageing female offspring exhibiting decreased cardiac output. Altered in vivo cardiac function in ageing female offspring may be linked to changes in cardiac oestrogen receptor expression.

## 1 INTRODUCTION

Cardiovascular disease (CVD), including coronary heart disease (CHD), stroke and vascular diseases are dominant contributors to morbidity and mortality worldwide (Groenewegen et al., 2020). Whilst there has been a substantial decrease in CHD mortality since the 1970s, this has begun to decelerate in countries around the world, including Australia (Mensah et al., 2017). This indicates that whilst reductions in modifiable risk factors (e.g., smoking) limit the burden of disease, additional factors nonetheless contribute to CHD. Both low weight at birth and low weight at 1 year of age have been associated with risk of death from CHD in adult life (Barker et al., 1989). Furthermore, being born small for gestational age, with accelerated growth in the proceeding 7 years, greatly increases the odds of death from CHD (Eriksson et al., 1999). Such observations led Barker and colleagues to propose that prenatal influences leading to low birth weight and accelerated postnatal growth could confer increased life‐long cardiovascular risk.

Animal studies have been employed to investigate influences of maternal perturbations on offspring cardiovascular health. A low protein diet during pregnancy has been shown to decrease rat offspring size, together with heart mass and cardiomyocyte endowment (Corstius et al., 2005). Functional deficits following maternal under‐nutrition in rats have been linked to reductions in heart relative to body weight in male offspring at 21 days, and an increased heart‐to‐body weight ratio and reduced ejection fraction in both male and female offspring at 22 months old (mo) (Rodriguez‐Rodriguez et al., 2017). Reduced nutrition throughout gestation in baboons leads to systolic and diastolic dysfunction in adult offspring, mimicking accelerated ageing (Kuo et al., 2017). Similarly, the offspring of rats with maternal diabetes exhibit reduced ejection fraction, decreased shortening fraction and diastolic dysfunction without changes to cardiac output (Mdaki et al., 2016). Animal studies collectively demonstrate that both nutrient excess and deprivation alter heart structure and function in mammalian offspring.

Studies of heart function have also employed *ex vivo* models to examine myocardial responses to relevant stressors (including ischaemia and stretch) in offspring exposed to prenatal insult. Rats subjected to 50% maternal protein restriction develop hypertension and impaired recovery from 30 min global ischaemia *ex vivo*, and the hearts of male (but not female) offspring exposed to in utero protein restriction exhibit reduced functional recoveries in reperfusion (Elmes et al., 2007). Similarly, maternal nicotine blunts functional recoveries from ischaemia in hearts of both male and female offspring, with evidence of depressed normoxic function in female offspring (Lawrence et al., 2008). In addition to functional deficits, perturbations throughout pregnancy have also been linked to increased cardiac fibrosis and collagen deposition, and many reported outcomes are sex specific (Elmes et al., 2007; Lawrence et al., 2008).

Whilst anatomical and physiological deficits throughout gestation are well studied, less is known regarding impacts of perturbations around the time of conception. Given that a large percentage of pregnancies are unplanned, many are exposed to stressors prior to pregnancy recognition, during the periconceptional period. In this regard, a recent report shows that up to 60% of pregnancies are exposed to alcohol during this period in Australia (McCormack et al., 2017). There is evidence alcohol exposure in the second half of pregnancy in sheep increases relative heart weight, and decreases cardiomyocyte endowment in association with increased cardiomyocyte cross‐sectional area and fibrotic markers in the ventricles of adult offspring (Goh et al., 2011). Rat offspring exposed to low‐dose ethanol throughout gestation demonstrate increased left ventricular wall thicknesses during diastole, evidencing hypertrophy and increased fibrosis (Nguyen et al., 2014). Currently, it is unknown if alcohol exposure during the periconceptional period will programme deficits in cardiac function in adult life, either in vivo or *ex vivo*. The aim of this study was to explore the effect of periconceptional alcohol exposure on heart growth, function and pathology in male and female offspring, and to investigate potential mechanisms underlying sexually dimorphic outcomes.

## 2 METHODS

### 2.1 Ethics statement

All experiments were performed at the University of Queensland and were approved by the University of Queensland Anatomical Bioscience Animal Ethics Committee (project numbers SMBS/022/12/NHMRC, SMS/467/14/NHMRC).

### 2.2 Animal model

Virgin female Sprague–Dawley rats were given free access to a liquid diet consisting of either a 12.5% v/v EtOH diet (PC:EtOH) or control diet from 4 days before mating to 4 days after mating. This represents a moderate, acute alcohol stressor, as previously described (Dorey et al., 2018, 2014; Gardebjer et al., 2015). The control diet contained Sustagen hospital formula (Mead Johnson Nutritionals, Auckland, New Zealand), reduced fat milk, corn flour and essential minerals (ferric citrate, copper II sulphate, selenium and magnesium sulphate). Previous experiments (Gardebjer et al., 2015) identified a reduced consumption of the liquid diet in EtOH‐exposed dams, and hence the energy density of the EtOH‐diet was increased and the ingredients were modified to ensure an equal amount of macro‐ and micronutrients (C diet composition: 11.3% fat, 17.0% protein, 68.2% carbohydrates, 7.7 MJ/kg; EtOH diet: 11.9% fat, 13.6% protein, 50.7% carbohydrates, 11.8 MJ/kg).

**FIGURE 1:**
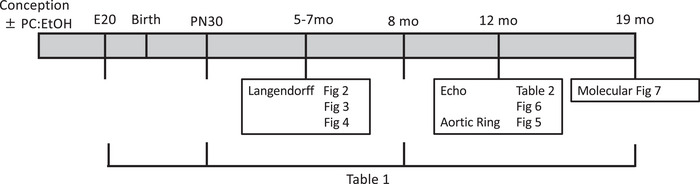
Overview of the study protocol. Females were exposed to periconceptual alcohol, and a subset of offspring were culled at embryonic day 20 (E20), postnatal day 30 (PN30), 8 months old (mo), 12 mo and 19 mo. Langendorff experiments including hyperaemia responses, pressure–volume and ischaemia–reperfusion were assessed at 5–7 mo, whilst aortic ring and echocardiography (Echo) were assessed at 12 mo.

A subset of dams were killed at day 20 of pregnancy (Gardebjer et al., 2014) for assessment of heart growth in utero. Remaining dams were placed onto a standard rat chow and offspring were weaned at 3 weeks, with two offspring cohorts produced. Cohort 1 consisted of *n* = 12 PC:EtOH dams and *n* = 12 control dams. One male and one female from each litter (maximum of 8 of each sex/treatment) were killed at day 30 and 8 mo for assessment of heart growth. No more than one pup of each sex per litter was used for each study parameter. Other offspring were used to examine aortic ring pharmacology and cardiac function in vivo at 12 mo before post‐mortem tissue collection at 19 mo. Cohort 2 was employed for *ex vivo* analyses of ventricular pressure–volume relationships, coronary reactive hyperaemia, and cardiac ischaemic tolerance at 5–7 mo, and consisted of *n* = 9 PC:EtOH dams and *n* = 8 Control dams. Figure 1 provides an overview of the treatment protocol and the timing of data acquisition as it relates to subsequent figures.

### 2.3 Animal growth and tissue collection

At embryonic day (E)20, dams were anaesthetised using 50:50 ketamine:xylazil (0.1 ml/100 g). At postnatal day (PN)30 and at 8 mo, a subset of offspring were anaesthetised using ketamine:xylazil (50:50, 0.5 ml/100 g BW), weighed and organs dissected. For E20 and PN30, given the low weight, a mean weight was determined for each sex, giving a single value representative of the litter. This single value is then used in the calculation of mean body weight of the control and PC:EtOH groups. At 19 mo, offspring were fasted overnight and anaesthetised with sodium pentobarbital (Lethobarb). The heart was excised and the left ventricle isolated, measured, snap frozen and stored at −80°C for mRNA and protein analysis. At 8 and 19 mo measurements of left ventricle thickness were made using callipers. Relative heart weight was calculated by normalising to body weight.

### 2.4 Echocardiography

Echocardiography was performed at 12 mo (*n* = 8 rats per sex per treatment group). Rats were weighed immediately prior to echocardiography. Anaesthesia was induced using isoflurane (5%) and maintained with 2% in 100% oxygen via a nose cone. Rats were laid supine on a heated pad and the thoracic wall shaved. Echocardiography was undertaken using a Philips HD15 ultrasound unit and high‐frequency (15 MHz linear array transducer) probe (Philips; Amsterdam, The Netherlands), and by a veterinary cardiologist as described previously (Nguyen et al., 2014). Measurements were taken in accordance with the American Society of Echocardiography using the leading‐edge method over three to five consecutive cardiac cycles minus mitral valve opening (MVc − MVo). Parameters measured included intraventricular septal width during diastole (IVSd), left ventricular internal diameter during diastole (LVIDd), left ventricular posterior wall at the end of diastole (LVPWd) and left ventricular internal diameter during systole (LVIDs). Heart rate was calculated from the R‐to‐R interval in consecutive QRS complexes and averaged over three intervals of a simultaneously recorded ECG. Doppler spectral profiles were used to measure isovolumetric relaxation time (IVRT), mitral valve closure and ventricular outflow allowing aortic ejection time (Ao ET), aortic velocity time integral (Ao VTI) and aortic cross‐sectional area (Ao CSA) to be measured. Using an established formula (Feigenbaum, 1989), fractional shortening (FS%) was calculated from LVIDd and LVIDs. The product of AoCSA, AoVTI and heart rate allowed calculation of cardiac output (CO). Myocardial perfusion index (MPI) was determined from the calculation:

MPI=[(MVc−MVo)−AoET]×AoET.

### 2.5 Plasma oestradiol levels

During post‐mortem at 19 mo, a cardiac puncture was performed on female offspring for the purposes of plasma oestradiol measurement. Whole blood was treated with heparin and plasma collected and stored at −20°C until analysis. Plasma oestradiol‐17β concentrations were measured using a commercially available radioimmunoassay (DSL4800, Ultra‐sensitive Oestradiol RIA, Beckman Coulter (Brea, CA, USA)); range 0; 5–570 pg/ml; intra‐assay CV < 9%, inter‐assay CV ≤ 12.2%), following the manufacturer's instructions.

### 2.6 Left ventricular gene and protein analysis

We also undertook an initial exploration of potential mechanisms behind the physiological outcomes. We chose to explore ventricular oestrogen signalling (given evidence of sex differences in physiological responses) and associated regulators of myocardial growth. We also selected a small suite of genes to test for possible shifts in regulation of tissue remodelling (e.g., *igf1*, *Agtr1a*, etc.; and IGF1R protein), and examined glucocorticoid and mineralocorticoid receptor mRNAs as a possible target of the Hsp90 changes revealed.

We assessed mRNA expression for oestrogen receptor α (*Esr1*; rn01640372_m1), heat shock protein 90 α (*Hsp90αα*; rn00822023_g1), insulin like growth factor 1 (IGF‐1; rn00710306_m1), angiotensin II receptor type 1 (*Agtr1a*; RN02758772_s1), glucocorticoid receptor (*Nr3c1*; rn00566193_m1) and mineralocorticoid receptor (*Nr3c2*; rn00585577_m1). Cellular RNA was extracted from left ventricular samples using RNeasy extraction kits (Qiagen, Hilden, Germany/Germantown, MD, USA) and cDNA synthesised using IScript reverse transcription kit (Bio‐Rad Laboratories, Hercules, CA, USA) as per the manufacturer's instructions. Data was analysed using the ∆∆*C*_t_ method with *Actb* used as endogenous control.

We further assessed ventricular protein expression of HSP90. Protein was extracted from left ventricular samples using lysis buffer (Cell Signaling Technology, Danvers, MA, USA). Protein concentrations were determined using the Bio‐Rad protein assay. Samples (20 µg) were loaded and subjected to SDS‐PAGE on 10% gels run at 130–180 V. Gels were transferred to membranes either for 2 h at 90 V or overnight at 30 V.

Odyssey buffer was used to block membranes followed incubation with a primary HSP90 antibody (C45G5, Cell Signaling Technology) applied overnight at 4°C, with a β‐actin antibody (BACT, 1:30000) added for an hour at room temperature. Membranes were washed with washing buffer (phosphate‐buffered saline with 0.1% Tween‐20) four times for 5 min. Secondary antibodies (LI‐COR IRDye 680 goat anti‐rabbit and IRDye 800CW goat anti‐mouse, Millennium Science, Mulgrave, Australia) were incubated for 1 h followed by 4 × 5 min washes. Protein expression was quantified using a LI‐COR Odyssey infrared imageing system (LI‐COR Biosciences, Lincoln, NE, USA).

### 2.7 Isolated heart perfusion (Langendorff) experiments

Offspring of 5–7 mo were anaesthetised with ketamine (10 mg/100 g) and xylazine (1.6 mg/100 g) until non‐responsive. The heart was excised and placed in ice cold buffer (modified Krebs–Henseleit buffer: 119 mM NaCl, 11 mM glucose, 22 mM NaHCO_3_, 4.7 mM KCl, 1.2 mM MgCl_2_, 1.2 mM KH_2_PO_4_, 0.5 mM EDTA, 1.85 mM CaCl_2_). The aorta was cannulated for retrograde perfusion of the heart using a Langendorff perfusion apparatus as described by us previously (Kaakinen et al., 2017; Reichelt et al., 2009). Perfusion pressure was maintained at 80 mmHg, and the perfusion fluid maintained at 37–37.4°C and bubbled with 5% CO_2_ in O_2_. The left atrial appendage was excised allowing insertion of a water‐filled balloon into the left ventricle, attached to a pressure transducer for data collection. The heart was then immersed in buffer within a water jacket temperature‐regulated water bath.

Two sets of experiments were performed in Langendorff perfused hearts: assessment of reactive hyperaemia and pressure–volume relationships in normoxic hearts, and assessment of intrinsic myocardial resistance to ischaemia–reperfusion.

### 2.8 Reactive hyperaemia and pressure–volume relationships

A subset of hearts (*n* = 7–9 per sex per group, 5–7 mo) underwent assessment of coronary reactive hyperaemia followed by examination of left ventricular pressure–volume relationships. Systolic pressure was set at 0 mmHg preceding a 20 min equilibration period. Coronary hyperaemic responses to a transient 30 s and 60 s occlusion were assessed, with 5 min reperfusion after each.

Hearts were then subjected to a further 15 min of normoxic perfusion during which they were paced at 300 beats/min and ventricular balloon volume adjusted to give a systolic pressure of 0 mmHg. After 15 min, the balloon volume was increased incrementally and function assessed over 2 min at each volume (7 initial increments of 10.1 µl, followed by increments of 20.2 µl until end‐diastolic pressure exceeded 25 mmHg). The slopes of the pressure–volume relationship for systolic and diastolic pressures were determined from the linear portions of the slope, from 0 to 50 µl/g.

### 2.9 Cardiac ischaemia–reperfusion

At 5–7 mo, a subset of experiments was performed to investigate intrinsic myocardial resistance to global ischaemia and reperfusion. Following 15 min equilibration, hearts were switched to pacing at 300 beats/min. After a further 15 min period, global no‐flow ischaemia was induced for 15 min followed by 60 min reperfusion. Pacing was stopped during ischaemia and re‐initiated at 2 min reperfusion. Post‐ischaemic data were normalised to pre‐ischaemic, paced values.

### 2.10 Aortic ring preparation

Following dissection, the thoracic aorta of 12 mo animals was placed into ice cold Krebs solution (118.0 mM NaCl, 4.7 mM KCl, 1.2 mM MgSO_4_, 1.2 mM KH_2_PO_4_, 2.5 mM CaCl_2_, 5.5 mM glucose, 25 mM NaHCO_3_) before removal of connective tissue and fat. Rings of ∼3 mm in width were mounted in an organ bath at one end connected to a pressure transducer and the other held stable within a heated bath of Krebs solution. This solution was gassed with a constant flow of 5% CO_2_ in O_2_ and maintained at 37°C. Rings were subjected to 1 g of initial tension and stabilised for 30 min. Maximum contractile force was determined by exposing rings to high K^+^‐substituted physiological salt solution (KPSS, 125 mM) containing equimolar substitution of KCl for NaCl. Vessels were then washed prior to a cumulative concentration–response curve to phenylephrine (1 × 10^−9^ to 3 × 10^−5^ M). Following washout, vessels were sub‐maximally contracted with a phenylephrine concentration that provided (60–80% of maximum KPSS) before constructing concentration–response curves to acetylcholine (ACh; 1 × 10^−9^ to 3 × 10^−5^ M) to assess endothelium‐dependent vasodilatation, and sodium nitroprusside (SNP; 1 × 10^−11^ to 3 × 10^−6^ M) to assess endothelium‐independent relaxation.

### 2.11 Statistical analysis

**FIGURE 2:**
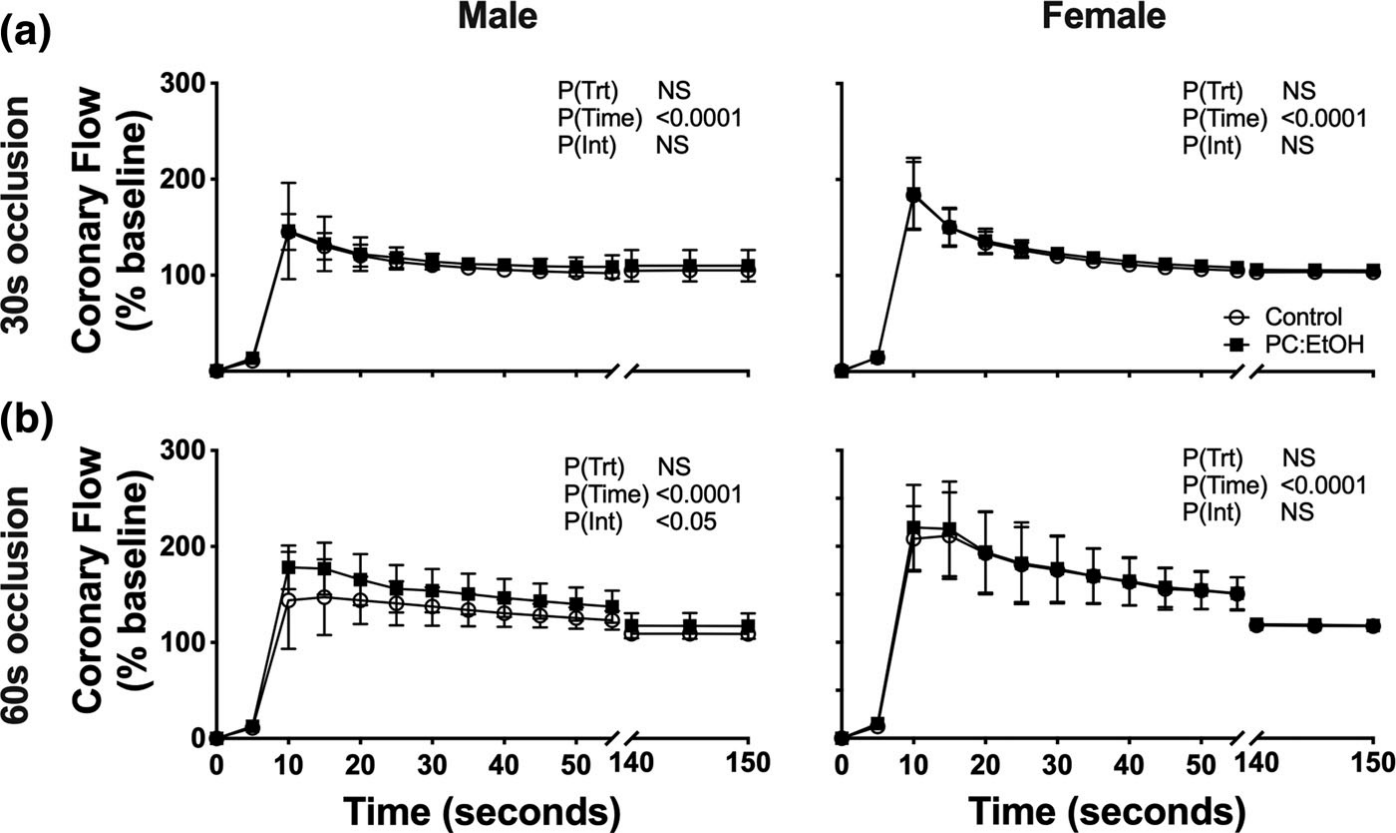
Coronary hyperaemic responses in perfused hearts from 5 to 7 month old rats subjected to a 30 s (a) and 60 s (b) occlusion. Responses are shown for hearts from male (left) and female (right) offspring from dams fed control (open circles) or PC:EtOH diets (filled squares). Flow responses are normalised to pre‐occlusion values. Data analysed by two‐way repeated measures ANOVA with Bonferroni post‐hoc analysis. Data are represented as means ± SD, ^*^ *P* < 0.05, ^**^ *P* < 0.01 versus control values at same time point, *n* = 6–9 per group.

**FIGURE 3:**
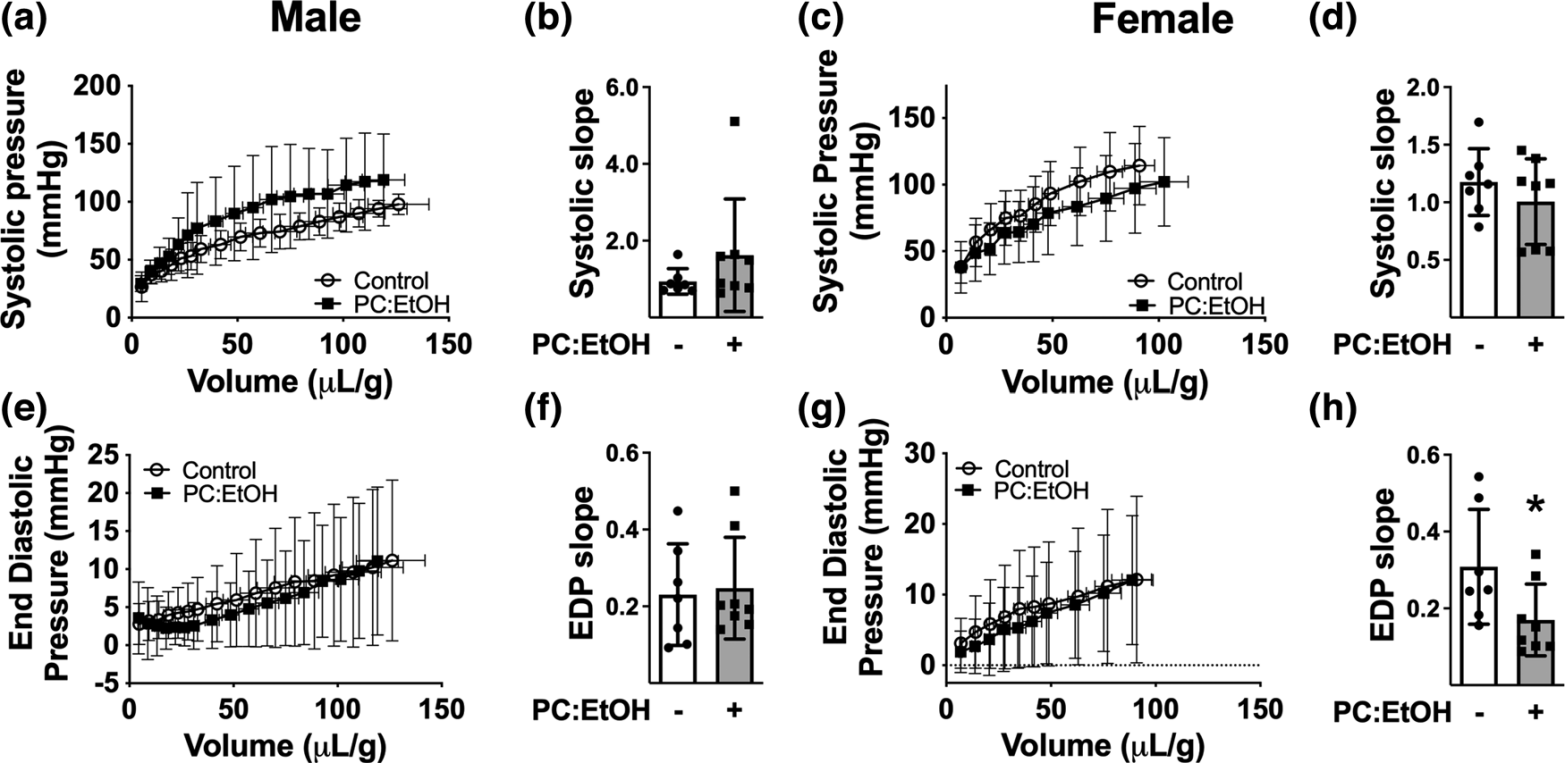
Systolic pressure (a, c) and end‐diastolic pressure (EDP) (e, g) changes during pressure–volume experiments in hearts from male (a, e) and female (c, g) offspring at 5–7 mo. The linear slopes for pressure–volume relationships (from 0 to 50 µl/g) are shown adjacent to each graph (b, d, systolic slopes in males and females, respectively; f, h, diastolic slopes for males and females, respectively). Ventricular volumes are normalised to total heart weight. Offspring from dams fed control (open circles) or PC:EtOH diet (filled squares). Data analysed by two‐way repeated measures ANOVA with Bonferroni post‐hoc analysis. Data are represented as means ± SD, *n* = 6–9 per group.

**FIGURE 4:**
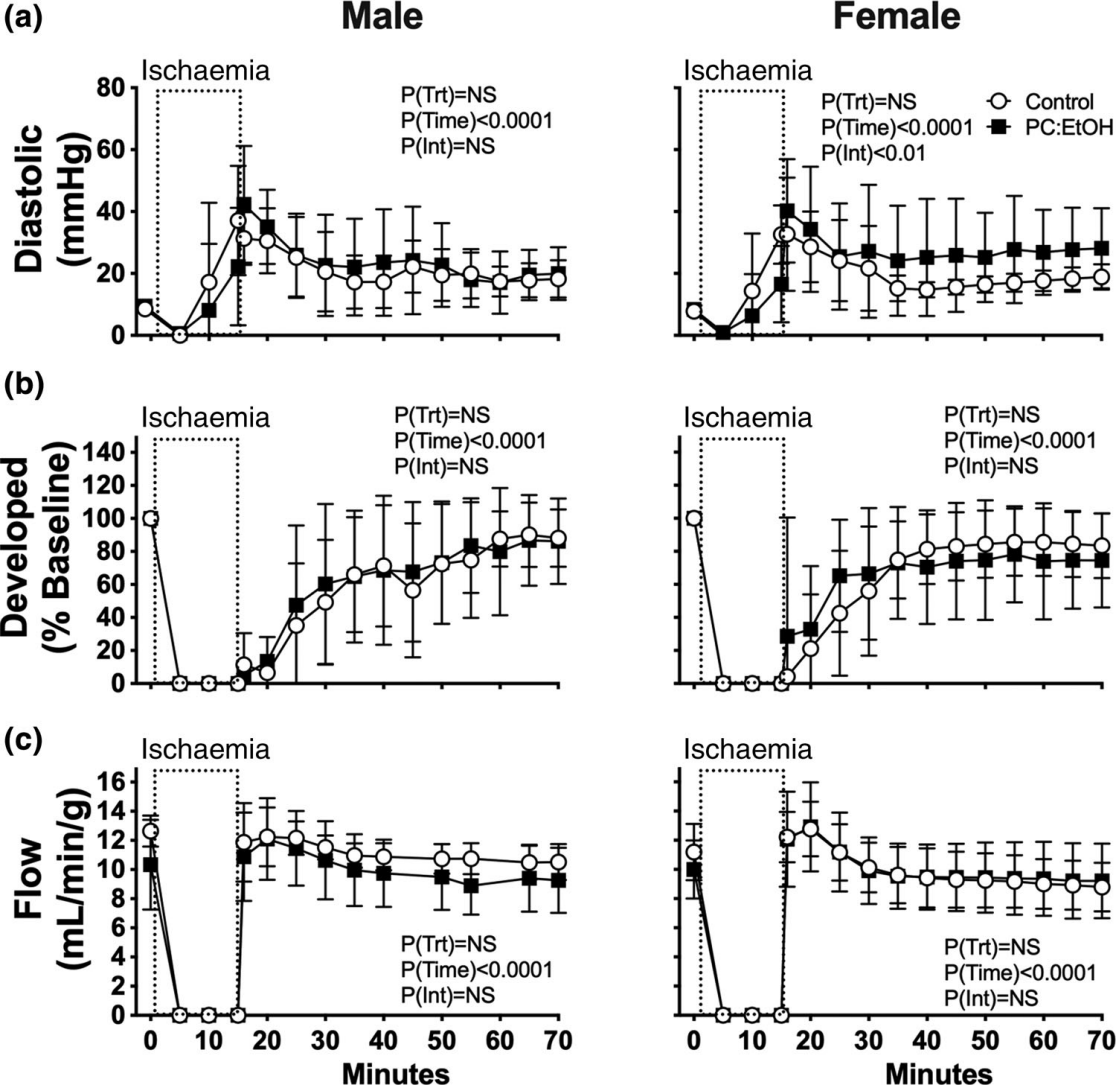
Functional changes during ischaemia–reperfusion in perfused hearts from 5–7 mo male (left) and female (right) offspring from control (open circles) or PC:EtOH dams (filled squares). Absolute changes in left ventricular end‐diastolic pressure (a) and responses for developed pressure (b) and flow (c) as percentage of pre‐ischaemia in Langendorff perfused hearts subjected to 15 min global ischaemia (dotted box). Data analysed by repeated measures 2‐way ANOVA with Bonferroni post‐hoc test. Data are means ± SD, *n* = 5–8 per group.

Organ weight and parametric gene expression data were analysed by two‐way ANOVA with sex and treatment as variables, with Bonferroni post‐hoc analysis where appropriate (Table 1, Figure 7a, f). Western blot (Figure 7d), echocardiography (Table 2, Figure 6) and aortic ring EC_50_ and Max data (Table 3) were analysed using Student's unpaired *t*‐test independently for each sex (Tables 1 and 3). Time‐course data from Langendorff experiments were analysed using repeated measures two‐way ANOVA with time and treatment as factors, with Bonferroni post‐hoc analysis (Figures 2 and 4). Where data did not meet the assumptions of a parametric test, a Mann–Whitney test (Figures 3b, d, f, h and 7c) or a non‐parametric Kruskal–Wallis ANOVA with Dunn's multiple comparisons test was performed (Figure 7b). Data are presented as mean ± standard deviation.

**TABLE 1:** Heart growth following PC:EtOH exposure.

	Male		Female		*P*		
	Control	PC:EtOH	Control	PC:EtOH	*P*_Trt_	*P*_Sex_	*P*_Int_
E20							
Heart weight (mg)	15.31 ± 1.07	14.89 ± 1.68	12.96 ± 2.92	14.38 ± 1.31	0.3974	**0.0202**	0.1253
Relative heart weight (mg/g)	5.76 ± 0.43	6.07 ± 0.44	5.14 ± 1.25	6.24 ± 0.62 **	**0.0049**	0.3547	0.1055
PN30							
Heart weight (mg)	389.92 ± 51.92	370.94 ± 44.46	369.53 ± 28.95	329.42 ± 45.59	**0.0437**	**0.0351**	0.4593
Relative heart weight (mg/g)	5.11 ± 0.30	5.02 ± 0.28	5.16 ± 0.39	5.03 ± 0.36	0.3124	0.7619	0.8435
8 Months							
Heart weight (g)	1.81 ± 0.26	1.69 ± 0.14	1.11 ± 0.15	1.05 ± 0.13	0.1147	**<0.0001**	0.5797
Relative heart weight (mg/g)	3.00 ± 0.44	2.80 ± 0.20	3.60 ± 0.94	3.44 ± 0.45	0.3473	**0.0021**	0.9242
LV thickness (mm)	3.50 ± 0.40	3.24 ± 0.42	3.01 ± 0.39	3.04 ± 0.41	0.3877	**0.0143**	0.2861
19 months							
Relative heart weight (g/g)	2.61 ± 0.32	2.75 ± 0.49	3.06 ± 0.60	2.98 ± 0.38	0.8367	**0.0044**	0.3126
LV thickness (mm)	3.72 ± 0.48	3.51 ± 0.76	2.86 ± 0.85	3.53 ± 0.44	0.3038	0.0628	**0.0479**

Data presented as mean ± SD. E20 and PN30, *n* = 9–11 litters; for PN 30 and E20 data, a mean weight was determined for each sex, giving a single value representative of the litter. Data analysed by 2‐way ANOVA with Bonferroni post‐hoc analysis. P_trt_, probability of treatement effect; P_int_, probability of interaction; *P = 0.05; **P = 0.01; ***P = 0.001. when compared control of same sex. *P*‐values shown in bold indicate statistical significance.

**TABLE 2:** Echocardiographic parameters comparing control and PC:EtOH offspring at 12 months of age.

	Male			Female		
	Control	PC:EtOH	*P*	Control	PC:EtOH	*P*
IVSd (mm)	1.71 ± 0.1	1.8 ± 0.2	0.3425	1.55 ± 0.2	1.45 ± 0.2	0.2598
LVIDd (mm)	8.55 ± 1.1	8.92 ± 0.3	0.3667	7.11 ± 0.5	7.28 ± 0.6	0.5547
LVPWd (mm)	1.78 ± 0.2	1.99 ± 0.4	0.1893	1.67 ± 0.1	1.56 ± 0.2	0.1191
Ao (mm)	3.82 ± 0.4	3.91 ± 0.2	0.6063	3.27 ± 0.2	3.13 ± 0.2	0.2163
Ao CSA	11.6 ± 2.4	12.05 ± 1.3	0.6521	8.42 ± 1.2	7.71 ± 0.9	0.1996
IVRT (ms)	28.4 ± 3.1	29.13 ± 3.3	0.6497	28.85 ± 4.7	27.45 ± 4.0	0.5305
MVc − MVo (ms)	106.5 ± 6.6	112.6 ± 12.6	0.2430	110.6 ± 8.2	109.1 ± 10.1	0.7638
MPI	0.27 ± 0.2	0.3 ± 0.1	0.6722	0.45 ± 0.1	0.44 ± 0.1	0.9659
Ao *V*_max_ (m/s)	118 ± 10.8	113.7 ± 11.4	0.5172	106.2 ± 10.4	98.84 ± 18.1	0.3343
Ao ET(ms)	84.9 ± 10.1	86.7 ± 5.8	0.6725	76.7 ± 8.1	75.61 ± 4.1	0.7346
Ao VTI (cm)	6.9 ± 0.6	6.9 ± 1.1	0.9584	5.7 ± 0.8	5.2 ± 1.4	0.3263
IVCT (ms)	21.9 ± 3.5	21.7 ± 2.0	0.9215	22.1 ± 2.7	20.1 ± 6.1	0.4074

Data presented as means ± SD, and analysed using Student's *t*‐test compared to same sex control. *n* = 8 per group. Abbreviations: Ao, Aorta; Ao CSA, aortic cross sectional area; Ao ET, aortic ejection time; Ao *V*_max_, aortic peak velocity; Ao VTI, aortic velocity time integral; CO, cardiac output; FS, fractional shortening; IVCT, isovolumetric contraction time; IVRT, isovolumetric relaxation time; IVSd, intraventricular septal width during diastole; LVIDd, left ventricular internal diameter during diastole; LVIDs, left ventricular internal diameter during systole; LVPWd, left ventricular posterior wall end diastole; MPI, myocardial perfusion index; MVc − MVo, mitral valve closure minus mitral valve opening.

**TABLE 3:** Tabulated results from aortic vascular reactivity in aortic rings from 12‐month‐old offspring.

			Male			Female		
			Control	PC:EtOH	*P*	Control	PC:EtOH	*P*
	PE (% KPSS)	pEC_50_	7.04 ± 0.34	6.73 ± 0.30	0.7221	6.99 ± 0.22	6.88 ± 0.27	0.4212
		Max	142 ± 34	127 ± 31	0.3643	120 ± 31	196 ± 114	0.0903
**Male**	ACh (% relaxation)	pEC_50_	7.29 ± 0.55	7.47 ± 0.21	0.4501	7.49 ± 0.48	7.07 ± 0.42	0.0928
		Max	100 ± 15	91 ± 10	0.2100	87 ± 21	105 ± 24	0.1514
	SNP (% relaxation)	pEC_50_	8.80 ± 0.20	8.65 ± 0.23	0.2242	8.75 ± 0.30	8.61 ± 0.38	0.5197
		Max	115 ± 11	107 ± 5	0.0929	114 ± 6	113 ± 4	0.7185

Data include negative logarithm of half‐maximal effective concentration (pEC_50_), and the maximal contraction (for PE) or relaxation (ACh, SNP) responses (Max). Data are means ± SD and are analysed via two‐way ANOVA with Bonferroni post‐hoc analysis with periconceptional treatment (Trt) and postnatal diet (Diet) as factors.

*n* = 6–8 per group.

## 3 RESULTS

### 3.1 Heart growth following PC:EtOH exposure

We have previously reported no impact of PC:EtOH on body weight, with the exception of lower weight in PC:EtOH embryos at E20 (Dorey et al., 2019; Gardebjer et al., 2018). At E20 there was no effect of PC:EtOH on average litter heart weight in the present study (Table 1), though female fetuses had lighter hearts than males (*P*_Sex_ = 0.0202). Together, this resulted in an increased heart weight relative to body weight following PC:EtOH (Table 1; probability of a treatment effect, *P*_Trt_ = 0.0049). At PN30, average heart weight was lower in the PC:EtOH group (Table 1; *P*_Trt_ = 0.0437) with hearts from female offspring weighing less than that of male offspring regardless of treatment (Table 1; *P*_Sex_ = 0.0351). However, relative heart weight (Table 1) did not differ between treatment groups at PN30. At 8 mo, PC:EtOH exposure did not impact heart weight, relative heart weight or left ventricle thickness (Table 1). Both heart weight and left ventricle thickness were lower in females compared to males (*P*_Sex_ = 0.0001 and *P*_Sex_ = 0.0143 respectively), whilst relative heart weight was higher (Table 1; *P*_Sex_ = 0.0021).

At 19 mo, PC:EtOH did not alter heart weight in offspring. Females had higher relative heart weights (Table 1; *P*_Sex_ = 0.0044) regardless of treatment. Interestingly, there was an interaction effect between sex and treatment in aged offspring for left ventricle thickness (Table 1; *P*_Int_ = 0.0479), and there was a 23% increase in the left ventricular thickness in the PC:EtOH females compared to control females.

### 3.2 *Ex vivo* heart function at 5–7 mo

To evaluate the function of the heart as a syncytium, Langendorff perfusion assays were performed. We focused on reactivity of coronary vessels, ventricular pressure–volume relationships and the cardiac response to ischaemia–reperfusion.

### 3.3 Reactive hyperaemia

To examine vascular reactivity, we used a short ischaemic challenge to cause a mild permutation of the balance between energy supply and demand. Following a 30 s ischaemic event, flow increased rapidly before declining to pre‐ischaemic values over the next 150 s (*P*_Time_ = 0.0001 for both Figure 2a, b). There was no effect of PC:EtOH in either male (Figure 2a left) or female (Figure 2a, right) offspring. Following 60 s occlusion, a more prolonged hyperaemic response was observed (Figure 2b), and an interaction between time and PC:EtOH treatment was detected for male offspring (*P*_Int_ = 0.0417; Figure 2b, left); however post‐hoc analysis revealed no significant PC:EtOH‐mediated change in flow (*P*_Trt_ = 0.073). PC:EtOH exposure did not affect the restoration of flow in the female offspring following the second occlusion (Figure 2b, right).

### 3.4 Ventricular pressure–volume relationships

Stretch dependencies of active force development (Frank–Starling) and diastolic force (stiffness or compliance) were assessed in hearts isolated from 5–7 mo rats. In both male and female offspring, increased balloon volume increased both systolic and diastolic pressures. PC:EtOH did not affect the systolic (Figure 3a, c) or diastolic (Figure 3e, g) pressure–volume relationship. The slope of the linear portion of systolic and end‐diastolic pressure curves was not altered by PC:EtOH in males (Figure 3b, f). The linear portion of the female systolic pressure was not modified by PC:EtOH; however PC:EtOH reduced the slope for the end‐diastolic pressure–volume relationship in hearts from female offspring (Figure 3h *P* < 0.05).

### 3.5 Response to ischaemia–reperfusion

Exposure to PC:EtOH did not alter baseline *ex vivo* cardiac function in offspring (Table 2). Diastolic dysfunction was evident in male and female hearts exposed to 15 min of ischaemia, and was unaltered by PC:EtOH (Figure 4a, right). Whilst ischaemia reduced developed pressure, there was no impact of PC:EtOH (Figure 4b). Initial recovery of coronary flow was slightly higher than pre‐ischaemic flow in the first 10 min of reperfusion, after which there was a decline to values that did not significantly differ from pre‐ischaemic flows, and this was unmodified by PC:EtOH in either males or females (Figure 4c).

### 3.6 Aortic ring function

**FIGURE 5:**
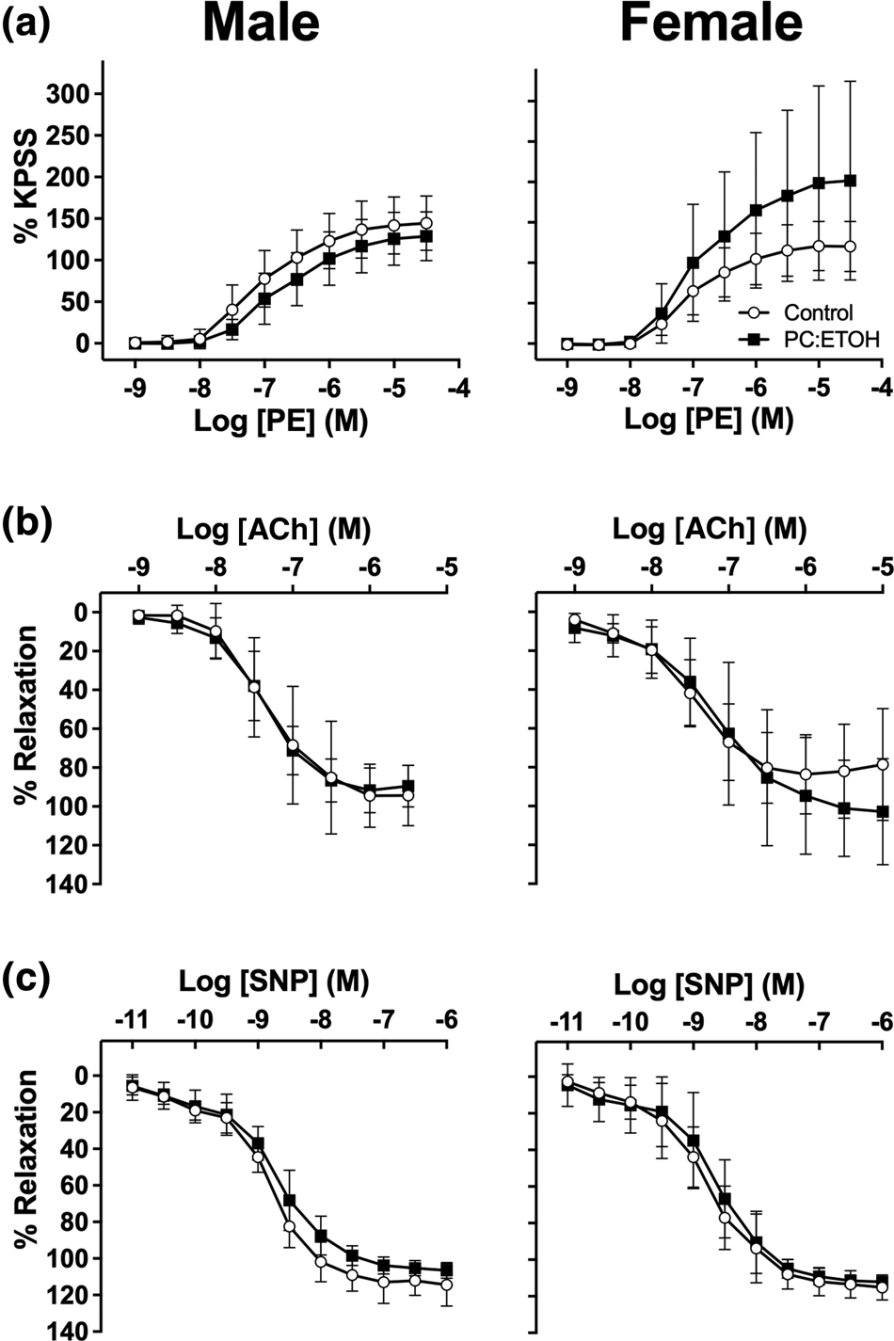
Vascular reactivity in 12 mo rat aortas. Phenylephrine (PE)‐induced contraction as a percentage of high K^+^‐substituted physiological salt solution (KPSS)‐induced contraction (a), together with acetylcholine (ACh)‐induced endothelium‐dependent (b) and sodium nitroprusside (SNP)‐induced endothelium‐independent (c) relaxations as a percentage of PE‐induced contraction in rings from male (left) and female (right) offspring during aortic organ bath experiments at 12 mo. Offspring are from dams fed Control white or PC:EtOH diets (black). Data are means ± SD, *n* = 6–8 per group. No significant differences were identified in EC_50_ or maximal responses.

Phenylephrine induced robust aortic constriction, which was not significantly modified by PC:EtOH in either males or females (Figure 5a), though there was a tendency to higher constriction across the concentration range in rings from females subjected to PC:EtOH (Figure 5a, right). Neither endothelium‐dependent relaxation to ACh (Figure 5b) nor endothelium‐independent relaxation with SNP (Figure 5c) was modified by PC:EtOH in either sex. Neither EC_50_ values nor maximal responses were modified by PC:EtOH (Table 3).

### 3.7 in vivo heart function at 12 mo

**FIGURE 6:**
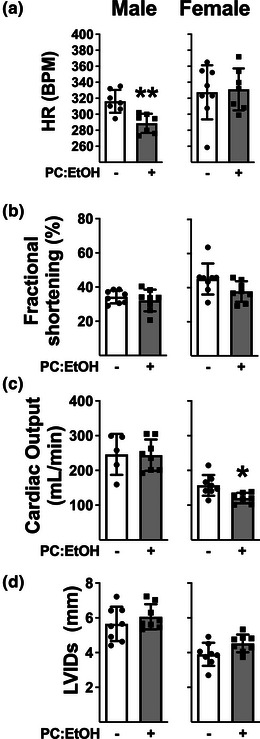
Key markers of cardiac function measured by echocardiography at 12 months of age in male (left) and female (right) offspring from dams fed a control (white) or PC:EtOH diet (grey). Heart rate (a), fractional shortening (b), cardiac output (c) and left ventricular internal diameter during systole (LVIDs, d). Data were analysed using Student's *t*‐test. Data are presented as means ± SD, *n* = 8 per group, ^*^ *P* < 0.05 when compared to controls of the same sex.

Due to large differences in body and absolute heart weight between male and female offspring, echocardiography data were analysed separately for each sex. In males, PC:EtOH decreased HR in anaesthetised offspring (Figure 6a, *P* = 0.0022), whilst in female PC:EtOH offspring, cardiac output was significantly reduced (Figure 6c; *P* = 0.0118). All other parameters were unchanged (Figure 6b, d, Table 2).

### 3.8 Oestrogen signalling and associated determinants of cardiac growth

**FIGURE 7:**
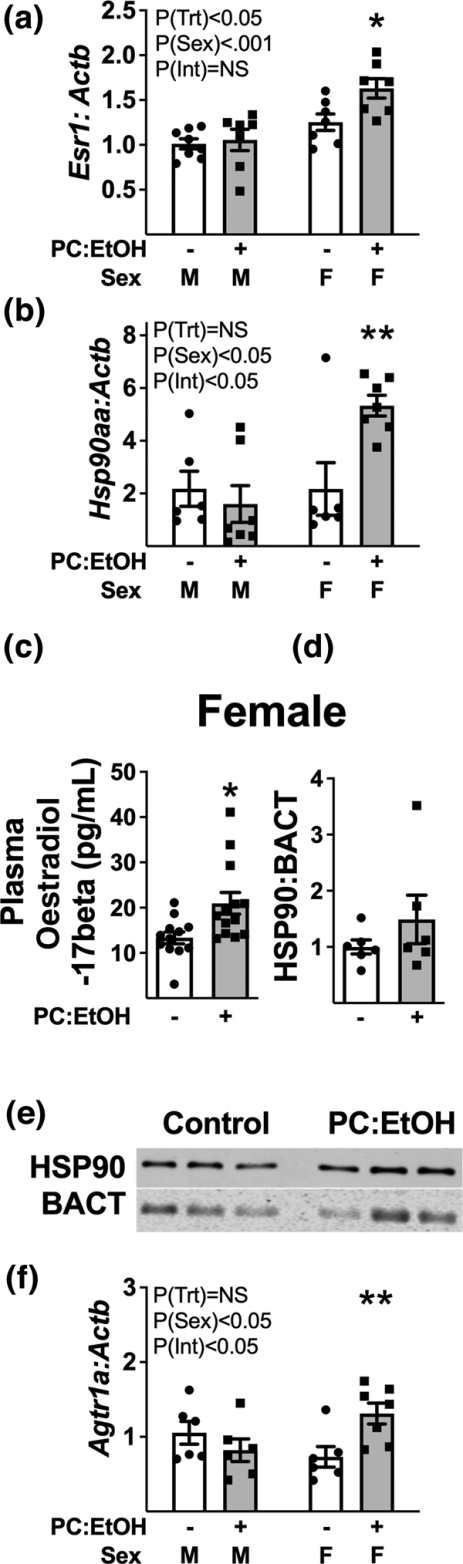
Cardiac oestrogen‐related factors and plasma oestradiol levels in 19 mo offspring from dams fed a control (white) or PC:EtOH (grey) diet. (a, b) Expression of mRNAs for oestrogen receptor α (*Esr1*, a) and heat shock protein 90 (*Hsp90aa*, b) in hearts of male and female offspring. (c, d) Plasma oestradiol‐17β levels (c) and left ventricular expression of HSP90 protein (d) in female offspring. (e) Representative western images for HSP90. (f) Expression of angiotensinogen type 1a receptor mRNA (*Agtr1a)* in hearts from male and female offspring. Data analysed by two‐way ANOVA, with Bonferroni post‐hoc analysis (a, b, f) or Students *t*‐test (c, d). Data are represented as mean ± SD, *n* = 6–8 per group. ^*^ *P* < 0.05, ^**^ *P* < 0.01 when compared to control of same sex.

Periconceptual EtOH increased cardiac expression of type 1 oestrogen receptor (ESR1) mRNA (Figure 7a; *P*_Trt_ = 0.043), with post‐hoc analysis identifying a select increase in female PC:EtOH offspring compared to female controls (*P* < 0.05). Expression of *HSP90aa* transcript was similarly modified in a sex‐specific manner: post‐hoc analysis also revealed increased expression in PC:EtOH female offspring when compared to control offspring of the same sex (Figure 7b, *P* = 0.039). Plasma oestradiol‐17β levels were also elevated in PC:EtOH female offspring compared to control female offspring at 19 mo (Figure 7c, *P* = 0.0152), whilst ventricular expression of HSP90aa was unaltered (Figure 7d). Female PC:EtOH‐exposed hearts also expressed higher *Agtr1a* transcript levels compared to control females (Figure 7e).

## 4 DISCUSSION

A recent Australian study found that 60% of women reported consuming alcohol between conception and pregnancy recognition (McCormack et al., 2017). Combined with high prevalence of CVD (Groenewegen et al., 2020), this highlights the importance of investigating how periconceptual events, such as maternal alcohol consumption, may programme heart disease in offspring. The present study is the first to demonstrate that exposure to only moderate volumes of alcohol, restricted to the immediate periconceptual period, can impact heart function in ageing female offspring. We show that periconceptional alcohol has sex‐specific effects on heart growth, and ageing female offspring exhibit decreased cardiac output. Interestingly, *ex vivo* analysis at 5–7 mo also reveals a decreased slope of the diastolic pressure–volume relation in this group (vs. controls), suggesting increased compliance. Altered in vivo cardiac function in female offspring may be linked to changes in cardiac oestrogen receptor expression.

Whilst relative heart weight in female fetuses (E20) was increased by PC:EtOH, this reflects maintained heart growth (‘heart sparing’) in the face of overall fetal growth restriction. However, by PN30 whole heart weight was lower in the PC:EtOH group, suggesting slowed heart growth in the early postnatal period, despite catch‐up growth. Transient fetal heart catch‐up growth has been observed with prenatal glucocorticoids (between E15 and E17.5), in association with increased transcripts for angiotensin II receptor type 1a (AT1aR), Bcl‐2‐associated X protein (BAX) and insulin‐like growth factor‐1 (IGF‐1) (O'Sullivan et al., 2013). Left ventricular hypertrophy was not evident in 12 mo echocardiographic analysis in the PC:EtOH group, though it is possible functional changes may emerge in older cohorts.

Up‐regulation of AT1aR transcript in the hearts of females with PC:EtOH exposure suggests activation of cardiac growth signalling, consistent, for example, with hypertrophy of post‐mitotic cardiomyocytes in compensation for perinatal growth restriction (which reduces cardiomyocyte endowment; Black et al., 2012). However, we do not detect an associated hypertrophic response at the ages assessed here. There is a paucity in the literature of links between periconceptional stressors and later life hypertrophy, but alcohol throughout gestation in rats has been shown to culminate in left ventricle hypertrophy by 8 mo (Nguyen et al., 2014). Although there is evidence that decreases in oestrogen may increase tissue *Agtr1a* expression (Nickenig et al., 1998), we observe parallel elevations in both oestrogen and *Agtr1a* in aged females exposed to PC:EtOH. Whilst speculative, this suggests PC:EtOH may disturb the regulatory influences of oestrogen on *Agtr1a* expression.

Decreased cardiac output is a feature of dilated cardiomyopathy. Characterised by left ventricle dilatation/enlargement and contractile dysfunction in the absence of a pressure or volume overload, dilated cardiomyopathy is the third most common cause of heart failure (Mathew et al., 2017). Though links between prenatal perturbations and dilated cardiomyopathy are unclear, dietary alterations can induce a dilated cardiomyopathy phenotype in murine models. Dietary selenium deficiency in rats induces left ventricular dilatation and systolic dysfunction (Shan et al., 2015). Cardiac functional deficits have also been recapitulated via cardiac‐specific knock out of oestrogen receptor β (ERβ), with changes closely resembling those following PC:EtOH exposure: increased left ventricle systolic and diastolic diameters with decreased fractional shortening (Rowe et al., 2017). This suggests oestrogen signalling might play some role in the phenotype emerging after PC:EtOH exposure, or could be up‐regulated in response to cardiac stress in an attempt to mitigate damage resulting from PC:EtOH. Cardiac tissue from patients with end‐stage dilated cardiomyopathy has increased mRNA and protein expression for oestrogen receptor α (ERα). These changes correlated with altered ERα distribution, with loss of characteristic co‐localisation of ERα/β‐catenin at the intercalated disc (Mahmoodzadeh et al., 2006). Whilst the cause of dilated cardiomyopathy cannot be determined in some individuals, evidence is mounting for both dietary and genetic influences.

Given that dilated cardiomyopathy and *Agtr1a* are influenced by oestrogen, we examined oestrogen receptors and related genes in the hearts of offspring. There are three characterised oestrogen receptors: ERα, ERβ and a less well characterised G protein‐coupled oestrogen receptor (GPER). ERβ was undetectable in aged female hearts in our model, consistent with previous studies (Tomicek et al., 2013). Interestingly, ERα mRNA was increased in the hearts of PC:EtOH female offspring, perhaps indicating a sensitivity to circulating oestrogen in these older animals. Oestrogen decreases with age in females of many species (Sokol et al., 1999), and oestrogen levels are thus predicted to be quite low in 19 mo offspring here. Furthermore, oestrogen supplementation decreases the ventricular hypertrophy with aortic constriction and ovariectomy in mice, additionally implicating ERβ in protection against hypertrophy (Babiker et al., 2006). Surprisingly, plasma oestrogen levels following PC:EtOH exposure in aged (19 mo) offspring were significantly higher than control. Whilst an interesting finding, it is important to note that values (∼20 pg/ml) were only 25% of those reported in rats at ∼9 mo (Sokol et al., 1999), and the difference between PC:EtOH and Control levels was minor (∼5 pg/ml). To better understand this phenotype it would be helpful to quantify circulating oestrogen in female offspring in earlier life (5–9 months).

ERα can act as a transcription molecule, exerting effects on oestrogen response elements or acting directly on target tissues. Heat shock protein 90 (encoded by *Hsp90aa*) maintains ERα in its active form (Fliss et al., 2000). Whilst PC:EtOH exposure increased both ERα and HSP90 in offspring, it is important to note that HSP90 is also an important transcriptional regulator for additional steroid receptors, together with glucocorticoid (*Nr3c1*) and the mineralocorticoid (*Nr3c2*) receptors (Echeverria & Picard, 2010). Importantly, however, we found PC:EtOH did not alter transcript levels for *Nr3c1* or *Nr3c2* in the hearts of offspring.

in vivo analysis showed that PC:EtOH decreased cardiac output in female offspring. Despite this in vivo change evident at 12 mo, *ex vivo* investigation revealed minimal influences on isovolumic cardiac function in female PC:EtOH offspring at 5–7 mo. Given changes in contractility identified via echocardiography, we predicted that pressure–volume relationships for isolated myocardium might differ for female PC:EtOH versus control offspring. However, systolic pressure–volume relationships were not modified with PC:EtOH, whilst the initial slope of the diastolic pressure–volume relationship appeared reduced in female offspring. The latter suggests a decrease in passive stiffness, but an altered chamber diameter could also contribute – whilst we normalise ventricular volume to overall heart mass, this might mask early shifts in chamber dimensions (though this would be inconsistent with later echocardiography data suggesting no dimension changes). It is also important to note that the *ex vivo* model assesses *isovolumic* contractile function, and not shortening and ejection properties. Pressure–volume relationships are not widely studied following prenatal perturbations, but maternal obesity in mice reportedly decreases cardiac function at 12 weeks of age in male offspring (females not studied), altering both systolic and diastolic function (Blackmore et al., 2014).

Coronary vascular function in perfused hearts appeared insensitive to PC:ETOH, with no significant changes in arterial contraction/relaxation or baseline coronary tone. Peak hyperaemia has been linked to the actions of both NO and K_ATP_ (Zatta & Headrick, 2005). A lack of change in isolated arterial responses to endothelium‐dependent and ‐independent stimuli suggests no change in vascular NO signalling. Interestingly, intrinsic ischaemic tolerance in offspring hearts also appeared insensitive to PC:EtOH. Prenatal hypoxia reportedly impairs functional recovery from 10 min ischaemia (Reyes et al., 2015), and prenatal nicotine from day 4 of pregnancy worsens post‐ischaemic diastolic dysfunction in hearts of both male and female offspring (Lawrence et al., 2008). Conversely, a low protein maternal diet is reported to improve the ischaemic tolerance of hearts of male offspring, potentially involving shifts in adrenoceptor, noradrenaline and dopamine levels (Ryan et al., 2012). Interestingly, nuclear ERα activation in endothelium may decrease injury from ischaemic events (Menazza et al., 2017). A detailed mechanistic understanding of the localisation of ERα in the hearts of PC:EtOH offspring, and whether increased expression in females might protect from further ischaemic damage, require further investigation.

In summary, findings in the present study indicate that PC:EtOH can have long lasting adverse effects on in vivo heart function in mature to aged female offspring. Interestingly, these functional alterations were associated with sex‐specific alterations in ventricular expression of oestrogen related genes. Given *ex vivo* heart function in younger animals was largely unaffected by PC:EtOH, the results suggest adverse effects may become more evident with age, or require systemic influences not present in isolated hearts. The specific influence of PC:EtOH in aged female hearts is of particular interest given the incidence of cardiovascular disease increases steeply in post‐menopausal women, and suggests that PC:EtOH can influence progression of age‐related heart dysfunction in a sex‐dependent manner.

## AUTHOR CONTRIBUTIONS

Emily S. Dorey, Karen M. Moritz, John P. Headrick and Melissa E. Reichelt conceived and designed the research. All authors contributed to the acquisition, analysis and interpretation of data. Emily S. Dorey drafted the manuscript, and all authors revised it critically and contributed intellectual content. All authors approved the final version of the manuscript and agree to be accountable for all aspects of the work in ensuring that questions related to the accuracy or integrity of any part of the work are appropriately investigated and resolved. All persons designated as authors qualify for authorship, and all those who qualify for authorship are listed.

## CONFLICT OF INTEREST

The authors declare no conflict of interest.

## Supporting information


Statistical Summary Document

Dataset

## Data Availability

Data supporting the findings of this study are available in the supporting documentation of this article.
